# Tincture of White Soap1Read before the medical section of the Philosophical Society of the University Virginia, May 10, 1909.

**Published:** 1909-11

**Authors:** F. P. Dunnington

**Affiliations:** Charlottesville, Va.; Professor of Analytical Chemistry, University of Virginia


### SELECTED ARTICLE

### Tincture of White Soap.[1]

By F. P. DUNNINGTON, Charlottesville, Va.
Professor of Analytical Chemistry, University of Virginia.

[*Old Dominion Journal of Medicine and Surgery, August, 1909.*]

IT is assumed that when a surgeon is washing up, in preparation for an operation, the aim is to thoroughly cleanse the skin and to remove all grease and other matters which are liable to entangle or harbor bacteria. For this purpose, soft soap is generally employed, either as such or as made up into tincture of green soap, or other similar alcoholic solutions.

Three objections to soft or green soap appear, viz.: (1) It carries much free alkali, which tends to roughen the skin; (2) it has a disagreeable odor, often masked by the addition of oil of lavender or carbolic acid; (3) it clings to the skin and cannot readily be so completely removed that no odor is left.

With the aim of preparing a liquid soap which will not carry these objections, I sought to substitute for this use white Castile or Marseilles soap. This soap is soluble in about 9 parts of cold water and in about 17 parts of alcohol, which solutions are far too dilute for the use in question.

"White soap," however, is soluble in about two parts of dilute alcohol, and the addition of a little ammonia further increases this solubility, so that with it we can obtain a solution containing as much true soap as is present in an equal volume of tincture of green soap. (Note that green soap, as sold, retains the glycerine of the oil and much water.)

---

1. Read before the medical section of the Philosophical Society of the University Virginia, May 10, 1909.

The name and formula proposed for this preparation is as follows:

TINCTURE OF WHITE SOAP.

| | | | For 1 gallon. |
|---|---|---|---|
| ℞ | Of white soap, Conti's ....... | 300 gram. | 1200 gram. |
| | Of ammonia stronger ........ | 25 c.c. | 100 c.c. |
| | Of alcohol .................... | 350 c.c. | 1400 c.c. |
| | Of water, dist. ............... | 325 c.c. | 1300 c.c. |

The specific gravity of this is 0.97, which is identical with that of tincture of green soap.

To make one gallon: mix the liquids for it in one gallon jar and then add the soap, previously cut into coarse shavings. Crowd all the soap into the jar and cover it with a glass plate. After 12 hours stir, and again stir after some hours. Allow to settle 12 hours and then filter, decant or syphon off the clear liquid into a can which may be kept closed. There will remain a few ounces retaining the impurities of the soap, which may be used for less particular washing. If this liquid is exposed to evaporation for a few hours, ammonia and alcohol escape, and a mass of the consistency of green soap will be obtained.

This form of soap alone has been employed by the surgeons in the University Hospital for the past year or more. While this preparation is free from the three above mentioned objections to tincture of green soap, it presents the additional advantage of costing but $1.50 per gallon and, if the alcohol be obtained tax free, the cost will be about 60 cents per gallon, while tincture of green soap will cost one dollar or more.

